# A Bifunctional Silicon Dielectric Metasurface Based on Quasi-Bound States in the Continuum

**DOI:** 10.3390/nano11092357

**Published:** 2021-09-11

**Authors:** Jianan Wang, Weici Liu, Zhongchao Wei, Hongyun Meng, Hongzhan Liu, Jianping Guo, Manxing Yang, Yongkang Song, Liujing Xiang, Zhenming Huang, Haoxian Li, Faqiang Wang

**Affiliations:** 1 Guangzhou Key Laboratory for Special Fiber Photonic Devices, Laboratory of Nanophotonic Functional Materials and Devices, School of Information and Optoelectronic Science and Engineering, South China Normal University, Guangzhou 510006, China; jiananwang007@163.com (J.W.); wzc@scnu.edu.cn (Z.W.); hymeng@scnu.edu.cn (H.M.); lhzscnu@163.com (H.L.); guojpgz@163.com (J.G.); manxingyang@163.com (M.Y.); 2018022090@m.scnu.edu.cn (Y.S.); xiang_liujing@163.com (L.X.); Huangzm6069@163.com (Z.H.); HaoXian_12@163.com (H.L.); 2 School of Engineering, Guangzhou College of Technology and Business, Foshan 528138, China; liuweici-2002@126.com

**Keywords:** all-dielectric metasurfaces, bound states in the continuum, extreme Huygens’ regime, chirality

## Abstract

Quasi-bound states in the continuum provide an effective and observable way to improve metasurface performance, usually with an ultra-high-quality factor. Dielectric metasurfaces dependent on Mie resonances have the characteristic of significantly low loss, and the polarization can be affected by the parameter tuning of the structure. Based on the theory of quasi-bound states in the continuum, we propose and simulate a bifunctional resonant metasurface, whose periodic unit structure consists of four antiparallel and symmetrical amorphous silicon columns embedded in a poly(methyl methacrylate) layer. The metasurface can exhibit an extreme Huygens’ regime in the case of an incident plane wave with linear polarization, while exhibiting chirality in the case of incident circular polarized light. Our structure provides ideas for promoting the multifunctional development of flat optical devices, as well as presenting potential in polarization-dependent fields.

## 1. Introduction

As an effective tool for manipulating electromagnetic wavefronts, metasurfaces composed of periodic meta-atom arrays have shown excellent performance and great potential in many fields [1,2]. Based on Mie resonances and lower ohmic loss than plasma metasurfaces, resonant metasurfaces with nano-structured medium are limited in size to the sub-wavelength level, and can control the electrical and magnetic polarization by adjusting the size parameters, in order to take better control of the amplitude, phase, and polarization of the incident wave [3,4,5,6]. In recent years, more and more studies related to metasurfaces have focused on how to make these structures possess a higher efficiency or quality factor, under the premise of realizing the original specific function [7,8]. Bound states in the continuum (BICs) can provide an appropriate approach to meet this need [9]. As a concept of quantum mechanics, BICs were first proposed by von Neumann and Wegner, in the 1930s [10]. Later, the concept was gradually applied to a wider range of physics, such as acoustics and fluid mechanics [11,12,13]. Until the last few years, BICs have gained increasing interest in the field of optics, due to their remarkable properties; namely, their high-quality factor [14,15,16,17,18]. According to different initiation mechanisms, BICs can be divided into symmetry-protected BICs [11,19] and “accidental” BICs [20]. The former is achieved by the symmetry of the structure, while the latter is achieved by destructive interference [21], such as Fabry–Pérot BICs [22,23] or Friedrich–Wintgen BICs [24,25]. For ideal BICs, on account of their not being coupled with the continuous wave, the energy is confined to the local field and cannot be leaked, which is also known as an embedded eigenvalue or embedded capture mode [9]. In practical applications, due to various losses or non-observability of the performance of a perfect BIC, structures are usually designed according to quasi-BICs (Q-BICs).

Polarization is one of the most important properties of electromagnetic waves. It is a hot topic to observe the performance of polarization of incident light through the design and parameter tuning of metasurface structural units [26,27,28,29]. Liu et al. [30] proposed a zigzag structure quasi-BICs Huygens’ metasurface—also known as an extreme Huygens’ metasurface—which can achieve wide phase modulation over a 2π range with an ultra-high quality factor, while keeping the transmittance close to 1 due to the spectrally overlapping of electric and magnetic polarization when the incident light is a plane wave. Extreme Huygens’ metasurfaces are able to maintain an ultra-low radiative decay rate on the basis of the metasurface, which can be applied to various fields such as pulse shaping, dispersion engineering, supercavity lasing, and non-linear meta-optics. Gorkunov et al. [31] paid attention to the maximum chirality of circular polarized light, and proposed a method to achieve circular dichroism based on their quasi-BICs structure, which has potential in polarization modulation devices, optical sensing, chiral light sources, and so on. Zografopoulos et al. [32] designed all-dielectric metamaterials with strong ring resonance for polarization beam splitting. These devices can perform their own polarization functions independently, but the single function may be a major limitation for the development of devices in the future. Multifunctional metasurfaces exhibit great potential in applications. For instance, based on the galinstan metasurface element, a multifunctional polarization converter with ultrabroad bandwidth and large angular tolerance was demonstrated for the first time, which can tune the incident electromagnetic waves into any desired polarization state [33]. The converter provides potential for various applications such as intelligent radar and quantum technology of light manipulation. By controlling the interference of the Mie resonance multipole, a polarization-sensitive dielectric membrane metasurface is able to switch from a highly reflective magnetic mirror to a transparent Huygens surface when the incident polarization changes, which contributes to developing an advanced dielectric metasurface and improving the performance of multifunctional flat-optics devices [29].

We are interested in the development of multifunctional metasurfaces based on Mie resonances, in order to overcome single function limitations. Multifunctional metasurfaces have great potential to broaden the application range of planar optical devices, being able to make contributions to the integration and miniaturization of components. As a feasible scheme, polarization-dependent metasurfaces have attracted our focus. We paid attention to the fact that both the extreme Huygens’ regime and metasurfaces with maximum chirality are polarization-dependent. In addition, they can be further connected through BICs.

In this paper, we propose a bifunctional dielectric metasurface based on Q-BICs, which is composed of amorphous silicon columns embedded in a poly(methyl methacrylate) (PMMA) layer. The metasurface is able to influence the polarization by adjusting the parameters at two wavelengths, and then achieve different functions at different polarized light incidences. More specifically, in the near-infrared band, the metasurface demonstrates the extreme Huygens’ effect with high quality factor for linear polarized incident lights. Meanwhile, in the vicinity of the red range, the metasurface appears to be chiral for circularly polarized incident lights. This structure indicates that, in a single structure, the interaction between light and matter shows its diversity, which is conducive to the development of optical devices with high efficiency, compactness, and miniaturization, and which also have certain application potential in sensing [34], phase modulation [35], polarization switches [36], and optical detection [37].

## 2. Model and Methods

**Figure 1:**
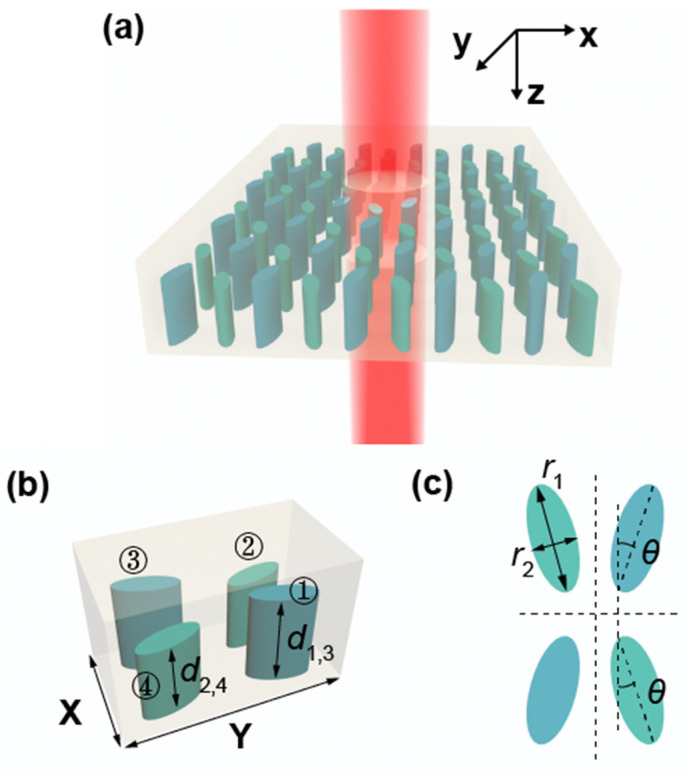
(**a**) Schematic of the proposed bifunctional metasurface composed of periodic arrays based on Q-BICs; (**b**,**c**) geometric description of a unit cell: X = 0.72 μm, Y = 1.3 μm, *d*_0_ = 0.52 μm, *d*_1,3_ = *d*_0_ + *D*/2, *d*_2,4_ = *d*_0_ − *D*/2, *r*_1_ = 0.265 μm, *r*_2_ = 0.0995 μm, *θ* = *πD*/*λ*.

The proposed periodic metasurface unit structure is shown in Figure 1. Asymmetric parameters are introduced and different phenomena are presented under different incident light, based on the Q-BICs mechanism. The structure consists of four elliptical silicon columns embedded in the PMMA layer. The elliptical cylinders are arranged in a zigzag shape, and there is an inclination angle of *θ*, with the vertical direction in the plane. It should be noted that the heights of the four elliptical cylinders in the unit are not all equal. The heights of elliptical cylinders 1 and 3 are *d*_1,3_ = *d*_0_ + *D*/2, while the heights of elliptical cylinders 2 and 4 are *d*_2,4_ = *d*_0_ − *D*/2, where the standard height is *d*_0_ = 520 nm and height difference *D* = *d*_1,3_ − *d*_2,4_. In order to achieve the maximum chirality, the angle *θ* and height difference *D* need to satisfy the following relationship: *θ* = *kD*/2 = *πD*/*λ*, where *k* and *λ* are the wave number and wavelength of incident circularly polarized light, respectively, when circular polarized light is incident.

For linear polarized waves incident in the normal direction along the z-axis, we consider that the optical response contains the incident and scattered fields in the direction perpendicular to the metasurface. The two effective collective mode resonances [30]—namely, electric polarization **P**(*ω*) and magnetic polarization **M**(*ω*)—constitute the expressions for the forward and backward scattered fields, **E**^f^ and **E**^b^:

(1) $$E^{f}=E^{i}+\frac{i\omega \eta }{2}(P+\frac{M}{c})$$

(2) $$E^{b}=\frac{i\omega \eta }{2}(P-\frac{M}{c})$$

where $\eta =\sqrt{\mu /\varepsilon }$ refers to the wave impedance of the background, and **E**^i^ refers to incident field. In fact, the ratio of **E**^f^ and **E**^i^ is equal to the transmission coefficient, while the ratio of **E**^b^ and **E**^i^ is equal to the reflection coefficient. As we can see from the formulas, the scattering fields in both directions mainly depend on the two polarizations. Taking this further, the transmission and reflection coefficients are contributed to by the electric and magnetic polarizations. In particular, the electric polarization **P** is determined by even polarity modes of electric fields along the Z-axis, while the magnetic polarization **M** is determined by odd polarity modes along the Z-axis. Furthermore, the dominant electrical polarization and magnetic polarization are arranged along the major and minor axes of the elliptical cylinders, respectively.

**Figure 2:**
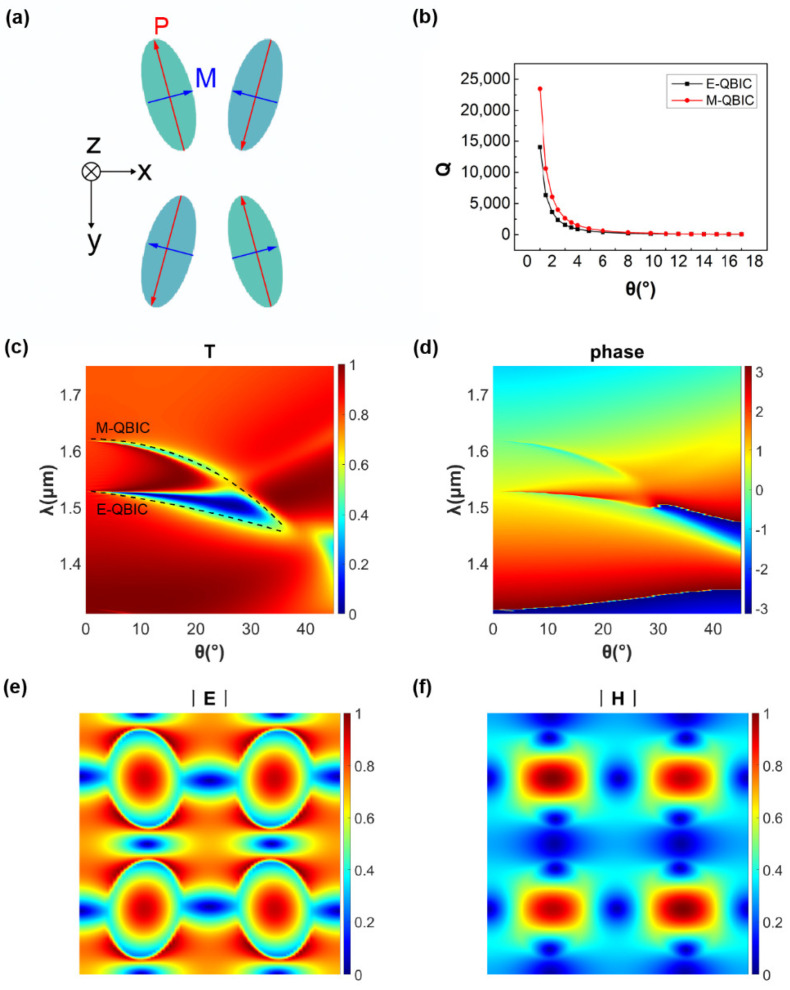
(**a**) Schematic of the concept of proposed extreme Huygens’ metasurface when linear polarized light is incident onto the metasurface; (**b**–**d**) simulation results of *Q* factors, transmission spectra, and phase spectra; and (**e**,**f**) distribution of electromagnetic field at the cross-section plane of *d*_0_/2 = 0.26 μm when *θ* = 3° and *D* = 12.28 nm.

For the general case, we assume that the incident field is linear polarized light, electrically polarized in the x direction and magnetically polarized in the y direction. On account of the symmetry of the structure, the plane wave is coupled to the collective mode with net polarization (*P_x_*, *M_y_*) after incidence. As shown in Figure 2a, when the inclination angle *θ* and height difference *D* are both small, the primary polarization in the zigzag metasurface is perpendicular to the incident field; that is, the dominant electrical polarization is along the Y-axis and the dominant magnetic polarization is along the X-axis (*P_n,y_* ≫ *P_n,x_*, *M_n,x_* ≫ *M_n,y_*). However, due to the antisymmetric distribution of the elliptical cylinders, the dominant polarization component in the unit of the structure is cancelled out internally: $P_{y}=\overset{N}{\underset{n=1}{\sum }}P_{n,y}=0$, $M_{x}=\overset{N}{\underset{n=1}{\sum }}M_{n,x}=0$. Therefore, the out-of-plane coupling between the dominant polarization and the plane wave with incident electric and magnetic polarizations along the y- and x-axes, respectively, is prevented, which greatly reduces the radiation loss of the structure. When *θ* = 0 and *D* = 0, the whole structure is symmetrically protected and presents perfect BICs. When the asymmetric parameters (referring to the inclination angle *θ* and height difference *D*, here) approach zero but do not equal zero, the two kinds of net polarizations Q-BICs are the so-called electric quasi-BICs (E-QBIC) and magnetic quasi-BICs (M-QBIC).

In order to analyze the mechanism and function of the metasurface further, we utilized the finite-difference time-domain (FDTD) method to simulate the metasurface. As shown in Figure 2b, the extreme Huygens’ metasurface has an ultrahigh quality factor *Q*, where *Q* is approximately inversely proportional to the square of the asymmetric parameters, which is a general property governed by the symmetric protected BICs [19]. More specifically, the relationship between the quality factor *Q* and resonance is given as $Q=\omega_{0}/2\gamma$, where *ω* is the resonance frequency and *γ* is the radiative leakage rate [9]. When the asymmetric parameter is 0, the electric polarization and magnetic polarization of adjacent columns in the unit cancel each other out, giving rise to non-radiative electric and magnetic BICs. At this point, the radiative leakage rate, *γ*, is 0, and the quality factor, *Q*, which is inversely proportional to it, is infinite. On the contrary, bringing in non-zero asymmetric parameters, the net electric and magnetic polarization, along the x- and y-axes, respectively, emerge, whose strength is proportional to the magnitude of the asymmetric parameters. In other words, an increase in the asymmetric parameters brings an increase in the radiative leakage rate, which results in a decrease in the quality factor *Q*.

We implement Huygens’ metasurfaces based on Schelkunoff’s equivalence principle [38]. In a free space, a Huygens’ metasurface divides it into two parts whose fields are independent of each other. In this case, the imaginary electric (**J**_s_) and magnetic (**M**_s_) surface currents are required to satisfy the boundary conditions at the discontinuities of the fields (where **n** is a unit vector perpendicular to the interface and pointing from one side of the metasurface to the other, and subscripts 1 and 2 distinguish the two parts of space):

(3) $$J_{s}=n\times \left(H_{2}-H_{1}\right),M_{s}=-n\times \left(E_{2}-E_{1}\right)$$

Instead of utilizing impressed sources [39], polarization currents excited by incident fields are regarded as the desired surface currents [40]. In order to describe the electromagnetic properties of Huygens’ metasurfaces, the electric admittance ${\bar{\bar{Y}}}_{sm}$, magnetic impedance ${\bar{\bar{Z}}}_{se}$, and the average tangential fields **E**_t,avg_ and **H**_t,avg_ at the interface are introduced.

(4) $$E_{t,avg}={\bar{\bar{Z}}}_{se}\cdot J_{s},H_{t,avg}={\bar{\bar{Y}}}_{sm}\cdot M_{s}$$

Then, based on the generalized sheet transition condition (GSTC) [41] and its incremental work [42], the normalized impedance and admittance were extracted from the complex reflection (*R*) and transmission (*T*) coefficients:

(5) $$Y=\frac{2\left(1-T-R\right)}{1+T+R},\;Z=\frac{2\left(1-T+R\right)}{1+T-R}$$

According to the above equations, when the electric admittance and magnetic impedance are pure imaginary and equal, the transmission coefficient tends to 1, and the phase transmission range of 2π can be achieved by adjusting the impedance. Significantly, the uniform electric admittance and magnetic impedance correspond to the overlap of electrical and magnetic dipole resonance spectra. The spectral overlap satisfies the first Kerker condition to suppress the backward scattering of incident light [43,44].

For the convenience of analysis, as shown in Figure 2c,d, we simulated the transmission spectrum and phase diagram in the presence of asymmetric parameters *θ* and *D*; note that the abscissa in the Figure 2b–d is *θ*, but *D* actually varies with *θ*, according to *θ* = *πD*/*λ*_0_, where *λ*_0_ = 736.617 nm, as shown in the following.

By tuning the asymmetric parameters, the spectral shift of Q-BICs resonances at different wavelengths will lead to their intersection in the spectral domain, resulting in spectral overlap at the intersecting points. This corresponds exactly to the equivalence of electric admittance and magnetic impedance in the previous paragraph. In the vicinity of the E-QBIC and M-QBIC overlap region, the transmittance tends to 1 with a huge quality factor Q, and the phase varies within a range of 2π, which fits the concept of the extreme Huygens’ regime [30]. Figure 2e,f show the distribution of electric and magnetic fields in the cross-section of the structure, and it is confirmed that the dominant polarization is perpendicular to the incident polarization.

Next, we discuss how to achieve chirality when the incident light is circularly polarized. Gorkunov et al. [31] proposed a method to achieve maximum chirality, based on phenomenological coupled-mode theory (CMT) [21,45,46]. CMT is utilized to analyze the transmission and reflection from two aspects: the background channel and the resonance channel. The resonance channel depends on the excitation and irradiation of the eigenstates carried by the structure. The transmission and the reflection coefficients can be expressed as follows:

(6) $$t_{R}=\tau -\frac{m_{+}m_{-}^{\prime }}{i\left(\omega -\omega_{0}\right)-\gamma_{0}}$$

(7) $$t_{L}=\tau -\frac{m_{+}^{\prime }m_{-}}{i\left(\omega -\omega_{0}\right)-\gamma_{0}}$$

where *τ* is the background transmission amplitude and *ω*_0_ is the resonance frequency. The damping rate is $\gamma_{0}=\gamma_{d}+\gamma_{r}$, where $\gamma_{d}$ and $\gamma_{r}$ are the dissipative and radiative parts, respectively. Both *ω*_0_ and $\gamma_{0}$ are unrelated to the helicity of incident wave. In Equations (6) and (7), *m* represents the coupling parameter between the eigenstates and the incident wave, where the subscript represents the rotation direction of the polarized light, and the presence or absence of the superscript represents the two sides of the metasurface, respectively. In this case, we regard *m*_+_ as the coupling parameter for right-handed circular polarized (RCP) light with forward incidence, while $m_{-}^{\prime }$ represents the counterpart for left-handed circular polarized (LCP) light incident on the other side. Through analysis [31] under the framework of CMT, it has been found that the realization of a metasurface with maximum chirality requires three conditions: firstly, the eigenstates are selectively coupled to circular polarized light (e.g., the eigenstates on the sides of the metasurface are uncoupled to the RCP waves); secondly, it completely absorbs the circular polarized light in the opposite direction; and, thirdly, the critical coupling regime [47] is satisfied—namely, $\gamma_{d}=\gamma_{r}$.

**Figure 3:**
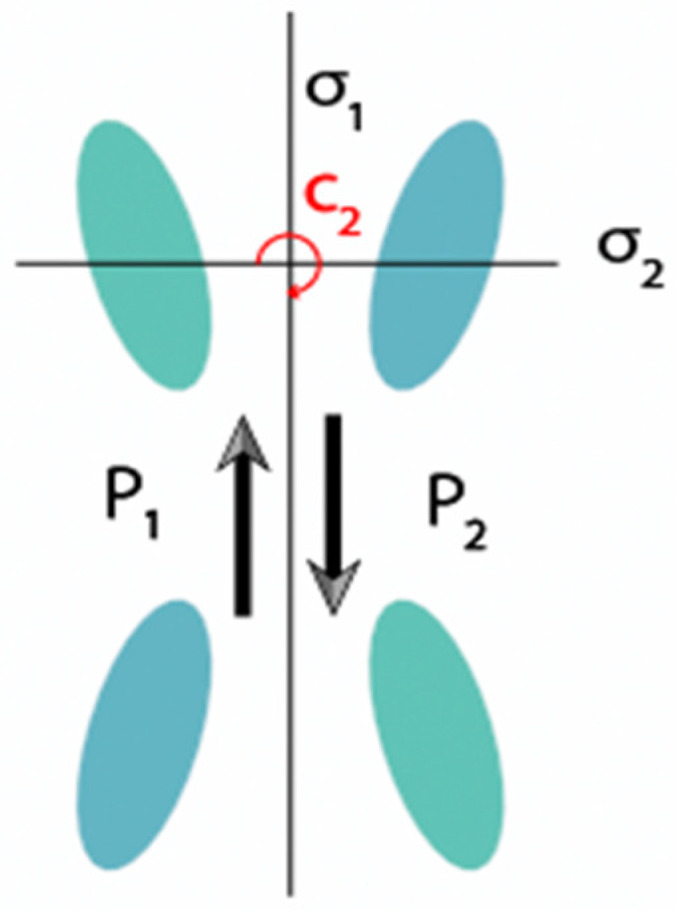
Schematic of the concept of maximum chirality, achieved when circular polarized light is incident onto the metasurface.

As shown in Figure 3, the electric resonance of the elliptical cylinders is described by a pair of antiparallel dipole moments **P**_1_ = −**P**_2_. By integrating the incident wave field with the eigenstate field (or, equivalently, with its current density), the coupling parameters between the incident light and the eigenstate can be approximately analyzed [19]:

(8) $$m_{e}\propto \int_{V_{1},V_{2}}dr\left[j\left(r\right)\cdot e\right]e^{ikz}$$

where the incident light is polarized along the unit vector **e** and is in possession of a wave vector **k** along the z-axis, while *V_1,2_* represents the volume of the two elliptical rods.

When the elliptical rods are completely symmetric, the dipole moment is in the symmetric protected BIC state, and the eigenstate is not coupled with any incident polarization:

(9) $$m_{e}\propto p_{1}\cdot e+p_{2}\cdot e=0$$

By breaking the symmetry of the structure, the dipole moment can be transformed into a Q-BIC. When there is a small asymmetric parameter *θ*, the mirror symmetry σ_2_ and rotational symmetry C_2_ of the unit structure are destroyed. Substituting $e=e_{\pm }$ into Equation (8), we have:

(10) $$m_{\pm }\propto p_{1}\cdot e_{\pm }+p_{2}\cdot e_{\pm }=i\sqrt{2}p\sin\theta$$

The presence of another small asymmetric parameter, *D*, destroys both the mirror symmetry σ_1_ of the unit structure and the rotational symmetry C_2_. In this case, the coupling parameter represents:

(11) $$m_{\pm }\propto p_{1}\cdot e_{\pm }+p_{2}\cdot e_{\pm }\cdot e^{ikd}=i\sqrt{2}pe^{ikd/2}\sin kD/2$$

It can be found that the structures, in both cases, are still chiral, as the three symmetries cannot be broken at the same time when only *θ* or *D* exists. When the asymmetric parameters *θ* and *D* exist together, all three kinds of symmetries are destroyed. In this case, the coupling parameter is:

(12) $$m_{\pm }\propto p_{1}\cdot e_{\pm }+p_{2}\cdot e_{\pm }\cdot e^{ikd}=i\sqrt{2}pe^{ikd/2}\sin\left(kD/2\mp \theta \right)$$

Then, the coupling parameter on the other side of the structure is:

(13) $${m^{\prime }}_{\pm }\propto i\sqrt{2}pe^{ikd/2}\sin\left(kD/2\pm \theta \right)$$

It can be found, from Equations (12) and (13), that when *θ* = *kD*/2 = *πD*/*λ* is satisfied, the maximum chirality can be achieved. At this time, the coupling parameters of RCP waves and the eigenstates on both sides of the structure,$\;m_{+}=m_{-}^{\prime }=0$, can be obtained. The coupling parameter of the other rotational direction—namely, LCP waves—is:

(14) $$m_{-}={m^{\prime }}_{+}\propto i\sqrt{2}pe^{ikd/2}\sin\left(2\theta \right)$$

Therefore, the critical coupling mechanism can be achieved by adjusting *θ* to achieve the true maximum chirality. It should be noted that the derivation of the above formulas referred to the work of Gorkunov et al. [31].

## 3. Results and Discussion

### 3.1. Extreme Huygens’ Metasurface Based on Q-BIC

**Figure 4:**
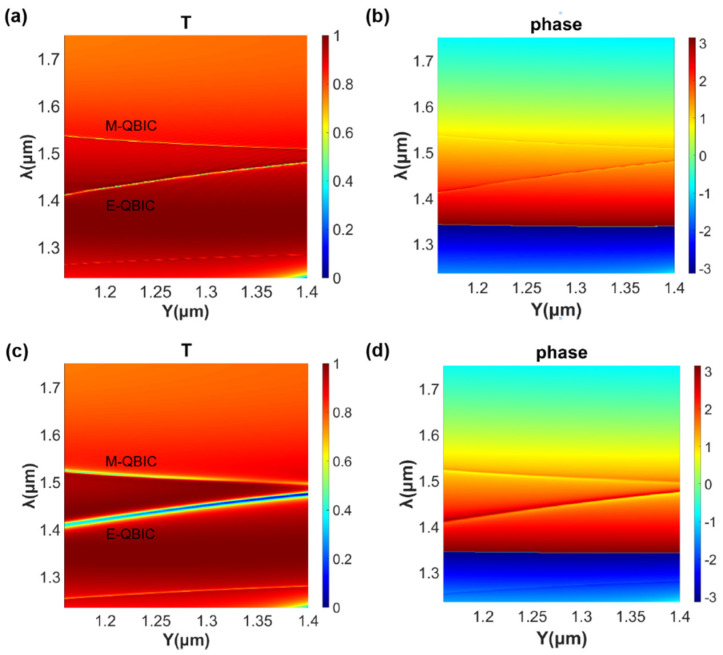
(**a**,**b**) Simulated transmission spectra and phase spectra as a function of the incident wavelength and the longitudinal period at the incidence of linear polarized lights when *θ* = 3° and *D* = 12.28 nm; (**c**,**d**) the counterparts of (**a**,**b**) when *θ* = 9.77° and *D* = 40 nm.

The proposed structure can realize the Huygens’ Q-BICs mechanism in the near-infrared band under the incidence of vertical linearly polarized light. Under the irradiation of a near-infrared broadband source, introducing asymmetric parameters *θ* = 3° and, correspondingly, *D* = 12.28 nm, the transmission amplitude and phase are shown in Figure 4a,b, respectively, as a function of the longitudinal period Y and wavelength. Meanwhile, Figure 4c,d represent the transmission amplitude and phase when *θ* = 9.77° and, correspondingly, *D* = 40 nm. The transmission amplitude of the metasurface for the incident light is rather close to 1 in the vicinity where the E-QBIC and M-QBIC tend to overlap, and the phase modulation over a 2π range can be achieved by adjusting the period Y. It can be seen that, with a decrease in the asymmetric parameters, the linewidth of the electric and magnetic Q-BICs becomes smaller, and the transmission amplitude in the neighborhood is closer to 1. When the asymmetric parameters are all zero—that is, when the symmetry protection mechanism is not destroyed—the spectra of the two quasi-BICs overlap, and the structure is in a perfect BIC. Different from Figure 2c,d, the structural period parameter, Y, is adjusted instead of the asymmetric parameters, such that the two Q-BICs tend to overlap. In this way, Schelkunoff’s equivalence principle is satisfied. Then, the Huygens’ regime is realized.

**Figure 5:**
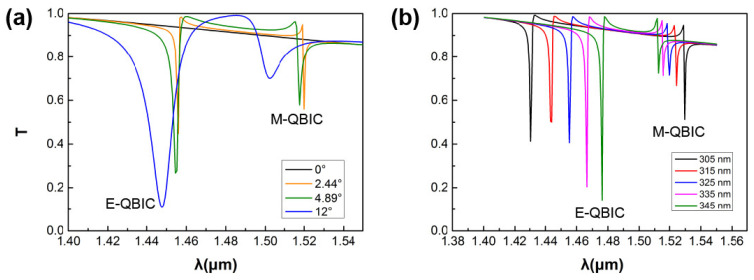
(**a**) The transmission phase spectra for different structural asymmetry parameters; and (**b**) the transmission spectra for varying longitudinal period Y under *θ* = 3° and *D* = 12.28 nm.

Figure 5a shows the variation of the transmission spectrum with *θ* and *D* in the presence of asymmetric parameters, from which the extreme Huygens’ regime can be seen more clearly. When the structure tends to be symmetrically protected—that is, when the asymmetric parameters become smaller and smaller—E-QBIC and M-QBIC tend to be numerically equal and closer to each other at the wavelength. Meanwhile, the line width of Q-BICS increases. Until there are no asymmetric parameters, the resonant modes cannot be observed, as the resonant modes are “dark’’ due to the protection of symmetry [48]. When *θ* = 3° and *D* = 12.28 nm, E-QBIC and M-QBIC approach as Y moves towards the longitudinal period at the intersection of the two BICs in Figure 4a, as shown in Figure 5b. In the presence of non-zero asymmetric parameters, we can observe two Q-BICs resonance modes with asymmetric linear shapes. As Y gradually increases within a certain range, the peaks of the two Q-BICs tend to be closer and equal, corresponding to the tendency of spectral overlap in Figure 2a. It can be seen that we can obtain the extreme Huygens’ regime by adjusting the sizes of the structure, including (but not limited to) the periodic parameter Y.

### 3.2. Chiral Metasurface Based on Q-BIC

When circularly polarized light is vertically incident onto the designed structure, the metasurface appears to be chiral. Through simulation, we found that the metasurface can perform differential absorption of RCP and LCP waves; that is, there is a certain circular dichroism.

**Figure 6:**
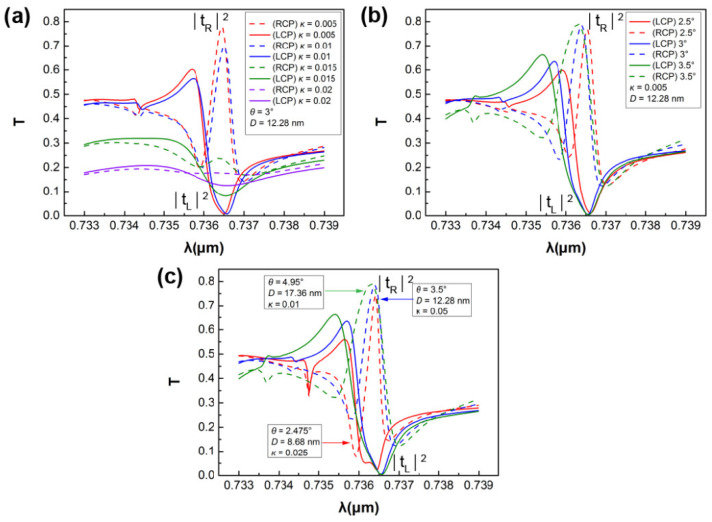
(**a**) RCP and LCP transmission spectra of chiral metasurfaces with different extinction coefficients and fixed asymmetry parameters: *θ* = 3° and *D* = 12.28 nm; (**b**) RCP and LCP transmission spectra of chiral metasurfaces with fixed height difference *D* = 12.28 nm and different rotation angles under the condition κ = 0.005; and (**c**) spectra of transmission of metasurfaces hosting maximum chiral quasi-BICs with symmetry-breaking parameters and losses, following the scaling rule $\theta^{2}\propto d^{2}\propto \kappa$. The colors in these three figures are used to indicate that curves are under different conditions, so that LCP and RCP curves under a same condition are in a same color. In order to distinguish the curves of RCP and LCP, we use solid lines to represent LCP and dash lines to represent RCP in all three figures here. According to our simulation and optimization scanning, we found that chirality of the metasurface can be achieved at *λ*_0_ = 736.617 nm. According to the conclusion derived from CMT, we control *θ* and *D* to satisfy *θ* = *kD*/2 = *πD*/*λ*, such that the initial maximum chirality is achieved. The remaining coupling parameters depend on damping which, in turn, is related to the extinction coefficient *κ*.

As shown in Figure 6a, setting *θ* = 3° and, correspondingly, *D* = 12.28 nm, we simulated the comparison diagram of the transmission of LCP and RCP light with different extinction coefficients *κ*. It was found that, when *κ* = 0.005, the maximum chirality was achieved. In addition, it can be seen that, at a slightly shorter wavelength, there was another resonance peak with low peak and broad line width, which is relative to magnetic-dipole resonance. Under the premise that other parameters are fixed—namely, *D* = 12.28 nm and *κ* = 0.005—the transmission spectra of the structure at different angles were simulated. The results are shown in Figure 6b, from which it can be observed that a better maximum circular dichroism could be achieved at *θ* = 3.5° than that at *θ* = 3°; that is, the uncoupling of the eigenstates from incident waves is ideal when *θ* = 3.5°. It was found that, on the basis of our theoretical calculations, the slight misalignment of the rod shape and the dipole moments results in a slight mismatch. From *θ* = *kD*/2 = *πD*/*λ* and Equation (14), we can obtain the general condition for achieving the maximum chirality: $\theta^{2}\propto d^{2}\propto \kappa$. To verify this rule, we simulated the transmission spectrum at *κ* = 0.0025 and *κ* = 0.001, as shown in Figure 6c.

To be sure, when achieving the maximum chirality, |t_R_|^2^ is around 0.8, instead of 1. In addition to inevitable structural losses, there is another reason, referring to the derivation in the previous section. First of all, we set the goal of making |t_L_|^2^ tend to 0 as much as possible, then the determined the relationship between *θ* and *D*. When a specific numerical *θ* or *D* is selected, *κ* is also determined, in order to achieve maximum chirality. It is important to note that *κ* is related to damping. For our structure, it fixes |t_R_|^2^, which reached a maximum of 0.8. In contrast, we can also set the goal of making |t_R_|^2^ infinitely close to 1 at first. However, |t_L_|^2^ will be much bigger than the original, instead of 0, correspondingly. In this case, both of the RCP and LCP waves can pass through the metasurface, which makes little sense, considering that we are searching for the maximum chirality.

For the proposed metasurface, |t_R_|^2^ cannot reach 1, but |t_L_|^2^ generally reaches 0. In this fashion, we can use our structure to realize the transmission of circular polarized light only in a single rotation. For the materials we used, the extinction coefficient at *λ* = 736.617 nm was 0.19, and the lower extinction coefficients (e.g., 0.005) could be obtained at higher wavelengths than 736.617 nm in the near-infrared band. Hence, a greater circular dichromatic resonance in the near-infrared range requires manufacturing technologies that can support precise nanoscale offsets.

**Figure 7:**
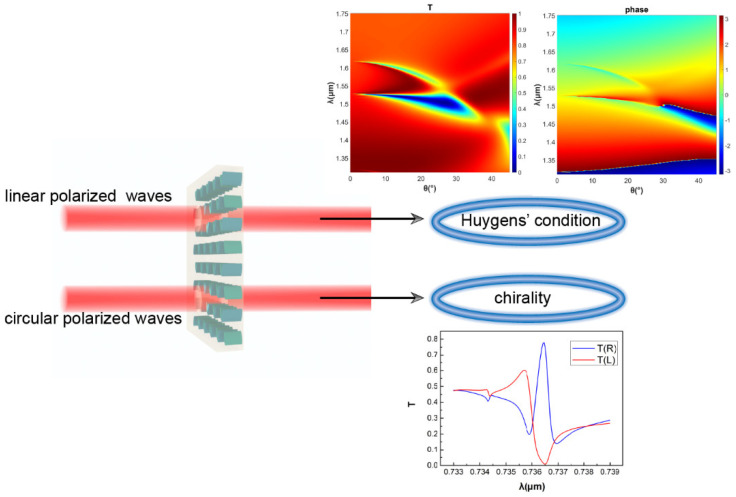
Schematic figure of the available functions that can be realized by the proposed metasurface.

To give a more general description of the dual functions of the proposed metasurface, Figure 7 presents a schematic diagram of the metasurface, where different incident light results in different effects. Under the perspective of similarities, both extreme Huygens’ regime and maximum chirality are polarization-dependent and, furthermore, are based on dielectric resonances, with multipoles making a contribution to the resonances. Moreover, Q-BICs are fulfilled by introducing perturbation to break the antisymmetric distribution of the structure for every function. From another point of the view, focusing on differences, the extreme Huygens’ regime appears in the near-infrared band with linear polarized light incident, and maximum chirality is achieved in the red band (at 736.617 nm, here) with circular polarized light incident. That is to say, the two functions require different polarization states and wavelengths of incident light, such that they will not affect the performance of each other. It is these similarities and differences that give rise to the desired metasurface.

## 4. Conclusions

In summary, we designed and simulated a polarization-dependent bifunctional resonant metasurface in a silicon medium based on the Q-BICs mechanism and Mie resonances. By introducing asymmetric parameters, we break the symmetry protection of the structure and realize the ideal Q-BIC. It can exhibit the extreme Huygens’ regime with high Q and a 2π phase modulation range at high transmittance, and it also can act as a chiral metasurface that allows only one direction of circularly polarized light to pass through, depending on the polarization of the incident light. According to Huygens’ condition, it was found that the metasurface can attain high dispersion. As a result of narrow resonance linewidth, it can be used as a supplement for optimization of existing, relatively wide-band devices, such as absorbers and optical communication devices. Moreover, the proposed structure is also helpful in the development of tunable metasurfaces, which can be applied to fields such as pulse shaping and dynamic polarization control. Furthermore, based on chirality, the proposed structure can be applied to the fields of photoelectric detection, optical switches, and non-linear optics. Combining the two functions, the metasurface, possessing the ability to efficiently regulate phase and polarization, is expected to be able to be widely used in holograms, polarization converters, sensors, and various industries.

We tactfully implemented two distinct functions on the same structure and well-maintained the performance of each, based on the use of BICs. The proposed metasurface provides a good platform to satisfy the demand of processing incident beams with different polarization separately. Devices are bound to develop toward high efficiency and miniaturization in the future. Our structure provides a feasible idea for the development of flat optical multifunctional devices with outstanding performance, integration, multi-functionality, and miniaturization.

## Data Availability

The authors confirm that the data supporting the findings of this study are available within the article. All data supporting the findings of this study are available from the corresponding author (J.W.) on request.
